# Hydrogen Promotes the Effectiveness of Bone Mesenchymal Stem Cell Transplantation in Rats with Spinal Cord Injury

**DOI:** 10.1155/2023/8227382

**Published:** 2023-05-04

**Authors:** Shengchang Luo, Jianxin Wu, Yuanyuan Qiu, Bing Xiao, Yanhai Xi, Chengwei Yang, Zhicai Shi, Weiheng Wang

**Affiliations:** ^1^Department of Orthopaedics, The First People's Hospital of Huzhou, No. 158, Plaza Back Road, Huzhou, 313099 Zhejiang Province, China; ^2^Department of Orthopaedics, The First Affiliated Hospital of Naval Medical University, No. 168 Changhai Road, Shanghai 200433, China; ^3^School Hospital of Shanghai University of Sport, No. 399, Changhai Road, Shanghai 200433, China; ^4^Department of Orthopaedics, The Second Affiliated Hospital of Naval Medical University, No. 415 Fengyang Road, Shanghai 200003, China; ^5^Department of Spinal Surgery, The 940th Hospital of Joint Logistics Support Force of People's Liberation Army, No. 333 South Binhe Road, Lanzhou, 730050 Gansu Province, China

## Abstract

Although bone mesenchymal stem cell (BMSC) transplantation has been applied to the treatment of spinal cord injury (SCI), the effect is unsatisfactory due to the specific microenvironment (inflammation and oxidative stress) in the SCI area, which leads to the low survival rate of transplanted cells. Thus, additional strategies are required to improve the efficacy of transplanted cells in the treatment of SCI. Hydrogen possesses antioxidant and anti-inflammatory properties. However, whether hydrogen can enhance the effect of BMSC transplantation in the treatment of SCI has not yet been reported. This study was aimed at investigating whether hydrogen promotes the therapeutic effect of BMSC transplantation in the treatment of SCI in rats. In vitro, BMSCs were cultured in a normal medium and a hydrogen-rich medium to study the effect of hydrogen on the proliferation and migration of BMSCs. BMSCs were treated with a serum-deprived medium (SDM), and the effects of hydrogen on the apoptosis of BMSCs were studied. In vivo, BMSCs were injected into the rat model of SCI. Hydrogen-rich saline (5 ml/kg) and saline (5 ml/kg) were given once a day via intraperitoneal injection. Neurological function was evaluated using the Basso, Beattie, and Bresnahan (BBB) and CatWalk gait analyses. Histopathological analysis, oxidative stress, inflammatory factors (TNF-*α*, IL-1*β*, and IL-6), and transplanted cell viability were detected at 3 and 28 days after SCI. Hydrogen can significantly enhance BMSC proliferation and migration and tolerance to SDM. Hydrogen and BMSC codelivery can significantly enhance neurological function recovery by improving the transplant cell survival rate and migration. Hydrogen can enhance the migration and proliferation capacity of BMSCs to repair SCI by reducing the inflammatory response and oxidative stress in the injured area. Hydrogen and BMSC codelivery is an effective method to improve BMSC transplantation in the treatment of SCI.

## 1. Introduction

The incidence of spinal cord injury (SCI) has varied from 14.6 to 60.6 per million in China in recent decades [1]. Traffic accidents and industrial accidents are the top two leading causes of SCI [2]. The treatment of SCI has always been a worldwide problem [3]. Traditional surgery and corresponding adjuvant therapy have not made breakthrough progress in SCI, causing serious family and social burdens [4]. In recent decades, the development of tissue engineering has provided prospects for the repair of the nervous system [5–7]. Stem cell transplantation can significantly improve neurological outcomes in experimental animals and clinical patients [8, 9]. The mechanism of stem cell transplantation for the treatment of SCI is complex and still unclear. Replacing damaged neuronal cells, reducing glial scar formation, and promoting axonal regeneration and synapse formation in residual neuronal cells are possible mechanisms [10]. All these possible mechanisms are crucially important for the repair of damaged nerve cells. Stem cell transplantation holds great promise for SCI treatment [11, 12].

Although stem cell transplantation holds great promise for SCI treatment, some hurdles need to be addressed to improve its efficacy. The microenvironment of the injured spinal cord is complicated and unbalanced [13]. Microenvironment imbalance of the injured spinal cord is defined as an increase in the inflammatory response [14] and oxidative stress [15, 16]. The microenvironment imbalance is the main cause of the poor regeneration and recovery of SCI, which also leads to low survival rates and poor viability of the stem cells transplanted into injured spinal cord areas [17]. Studies have shown that the acute phase of SCI is the best period for stem cell transplantation [18, 19]. Imbalanced microenvironments (inflammatory response and oxidative stress) were the most severe during this period, which led to low cell viability and insufficient secretion of the transplanted stem cells [16]. The main methods of cell transplantation for the treatment of SCI include spinal cord local injection, intrathecal transplantation (ICT), and intravenous injection [20, 21]. Despite the different transplantation routes, the cell viability was low and could not survive for a long time. How to improve the transplanted cells' survival and viability is the key problem to be solved.

Hydrogen (H_2_) therapy has attracted extensive attention due to its antioxidant and anti-inflammatory effects [22]. H_2_ possesses excellent permeability and biosafety and has been shown to attenuate intracellular reactive oxygen species- (ROS-) induced cytotoxicity and inflammatory responses [22]. H_2_ has shown good therapeutic effects in the treatment of various diseases, such as diabetes, sepsis, atherosclerosis, hypertension, and cancer [23–25]. H_2_ direct inhalation and hydrogen-rich water have been used in the treatment of SCI with good therapeutic effects [26–30]. H_2_ can improve the microenvironment imbalance by suppressing inflammatory responses and oxidative stress levels in SCI areas. Thus, we hypothesized that hydrogen can enhance the effect of BMSC transplantation in the treatment of SCI, which has not yet been reported. In this study, we confirmed the hypothesis that H_2_ can promote the effect of BMSC transplantation in the treatment of SCI by reducing the inflammatory response and oxidative stress.

## 2. Materials and Methods

### 2.1. Preparation of Hydrogen-Rich Saline (HRS) and Hydrogen-Rich Cell Culture Medium (HRM)

HRS and HRM were prepared according to the method reported previously [31, 32]. High-purity hydrogen (99.99%) was slowly poured into the normal saline and cell culture medium, and the pressure reached 0.4 MPa for 6 h. HRS and HRM were prepared. The H_2_ content was confirmed using the method described by Ohsawa et al. [22]. The H_2_ concentration of HRS and HRM was maintained above 0.6 mmol/L. Fresh HRS and HRM were produced weekly and stored in a refrigerator at 4°C.

### 2.2. Animals

All animal experiments were reviewed and approved by the Ethics Committee of Experimental Animal Management of the Naval Medical University. Female Sprague Dawley (SD) rats (180–220 g, about 8 weeks) and transgenic SD rats expressing green fluorescent protein (GFP) were purchased from the Experimental Animal Center of the Naval Medical University. The rats were housed in an animal room (5/cage, 20–22°C, 12 h light/dark cycle, 50–60% relative humidity) and had ad libitum access to food and water for 1 week prior to the experiment to adapt to the environment. SD rats were used for animal experiments. GFP rats were used to extract GFP-labeled bone marrow mesenchymal stem cells (BMSCs-GFP) for in vivo transplantation cell tracking.

### 2.3. Culture and Characterization of BMSCs and BMSCs-GFP

The culture and characterization of BMSCs and BMSCs-GFP were performed as we had previously reported [33]. SD rats were killed via CO_2_ asphyxia and sterilized with 75% alcohol for 10 min, and the bilateral femur and tibia were taken out aseptically. Both ends of the epiphyseal plate were severed. DMEM (Gibco; Thermo Fisher) was used to irrigate the marrow cavity. The cells were collected and seeded in a 10 ml T75 flask (Corning) and cultured in a 5% CO_2_-saturated humidity incubator at 37°C. After 24 h, the culture medium was replaced, and the medium was then replaced every 2 days. The CD90, CD105, CD73, CD45, CD34, CD11b, and CD19 antibodies (Guge, Nanjing, China) of cells were tested for cell purity via flow cytometry (FAC500, Beckman Coulter, USA) [34, 35]. Adipocyte, chondrocyte, and osteocyte differentiation media (ScienCell, San Diego, California, USA) were mixed with the medium that was replaced every 3 days. After 3 weeks, the cells were fixed and stained with Alizarin red, Oil Red O, and Alcian blue (Sigma-Aldrich, St. Louis, MO, USA) to examine their osteogenic, lipogenic, and chondrogenic properties, respectively. The cell morphology was evaluated under an optical microscope (Olympus, Japan) and fluorescence microscope (Leica, Germany).

### 2.4. The Effect of H_2_ on the Proliferation of BMSCs Detected via CCK-8 and EdU Staining

The effect of H_2_ on the proliferation of BMSCs was detected via the CCK-8 and EdU staining methods. P3 BMSCs were cultured in normal medium and HRM for 6, 12, 24, and 48 h. The CCK-8 kit (Biyuntian, China) was used in strict accordance with the detection steps in the kit instructions, and a microplate reader (BioTek, USA) was used to detect the proliferation of BMSCs via H_2_ at an ultraviolet wavelength of 450 nm. After P3 BMSCs were cultured in a normal medium and HRM for 48 h, BMSCs were incubated in a medium with a 20 *μ*M EdU working solution for 6 h and then were incubated with an EdU fluorescent solution for 30 min in the dark. The percentage of EdU+BMSCs was calculated based on images obtained using fluorescence microscopy. The experiment was repeated three times.

### 2.5. The Effect of H_2_ on the Migration of BMSCs Detected via the Transwell Assay

In this experiment, a Transwell assay (pore size 8.0 *μ*m) was used to detect the effect of H_2_ on the migration of BMSCs. 5 × 10^4^ cells/well of BMSCs were seeded in the upper chamber of the Transwell assay (Millipore, USA, 8.0 *μ*m), and 600 *μ*l of normal medium and HRM was added to the lower chamber. The Transwell assay was tested after 6, 12, and 24 h. Cells were stained with 0.1% crystal violet (Sevier, Wuhan, China) for 20 min and then captured with a microscope (Olympus, Japan). The experiment was repeated three times.

### 2.6. The Effect of H_2_ on the Tolerance to Serum-Deprived Medium (SDM) of BMSCs Detected via Flow Cytometry

The effect of H_2_ on the apoptosis rate of BMSCs and tolerance to SDM was detected via flow cytometry. P3 BMSCs were cultured in a normal medium and HRM for 12 h. The apoptosis of adherent cells was detected via flow cytometry. The Annexin V-PI Apoptosis Detection Kit (BD, USA) was used in strict accordance with the operation steps of the kit, and the effect of H_2_ on apoptosis was detected via flow cytometry (Beckman Coulter FC 500, USA). All staining procedures were completed according to the flow antibody instructions. The experiment was repeated 3 times.

### 2.7. Establishment of the Rat Model of SCI and H_2_-BMSC Administration

A total of 60 SD female rats (180–220 g) were used to establish SCI models. The SCI animal model was established using the classical vertical blow method [36, 37]. Rats were randomly divided into 4 groups: the sham group, which underwent laminectomy only (*n* = 15); the SCI group, which received intraperitoneal normal saline every 24 h (5 ml/kg, *n* = 15); the BMSC group, which received BMSCs and intraperitoneal injection of saline every 24 h (5 ml/kg, *n* = 15); and the H_2_-BMSC group, which received BMSCs and intraperitoneal injection of HRS every 24 h (5 ml/kg, *n* = 15). After general anesthesia, the rats were fixed in a Jiangwan type II rat immobilizer. The skin, spinous process, and lamina were incised to fully expose the spinal cord of the T10 segment. A 10 g metal rod was allowed to free fall from a height of 50 mm, and the spinal cord of the T10–12 segment was impinged upon. The rat tail and lower limb spasm during the operation indicated that the modeling was successful. Penicillin (500,000 units/intramuscular injection) was administered to prevent infection. The postoperative abdominal massage was performed (12 h/time) to assist in urination until the rats could urinate on their own. ICT was used for BMSC transplantation following SCI immediately at lumbar vertebrae L3–5 [38]. BMSCs (1 × 10^6^) suspended in 20 *μ*l of PBS were injected over 5 min to prevent cell leakage.

### 2.8. Functional Behavior Evaluation and CatWalk Gait Analysis

The Basso, Beattie, and Bresnahan (BBB) grading [39] was used to observe the functional recovery of lower limbs in the 4 groups (*n* = 10) before and 1, 3, 7, 10, 14, 21, and 28 days after SCI. Footprint analysis was performed as previously reported in the literature 28 days after SCI [30]. 28 days after SCI, CatWalk gait was used to evaluate the function of the hindlimbs in detail [40].

### 2.9. The Effect of H_2_ on the Survival and Migration of Transplanted BMSCs

Three and 28 days after SCI, the spinal cord at 3 mm in the center of the injured spinal cord was harvested (*n* = 5). The spinal cord tissues were cryosectioned (10 *μ*m), stained with hematoxylin-eosin (HE), and observed under an optical microscope (Olympus, Japan). Sections were stained by 4′,6′-diamino-2-phenylindole (DAPI) (Harvey, USA). The transplanted BMSC-GFP observed under a fluorescence microscope showed spontaneous green fluorescence. The sections were examined under a fluorescence microscope (Leica, Germany). The GFP-BMSCs were detected and observed. Ten fields were randomly selected from each group under a high-power field, and the number of positive cells and the distribution of cells were calculated.

### 2.10. The Effect of H_2_-BMSCs on Spinal Cord Inflammatory Factors (TNF-*α*, IL-1*β*, and IL-6) Detected via Enzyme-Linked Immunosorbent Assay (ELISA)

SCI rats were sacrificed 3 and 28 days after SCI, and the lamina was opened rapidly. The spinal cord in the region 3 mm from the center of the injured spinal cord was harvested, frozen in liquid nitrogen, and placed in a −80°C freezer (*n* = 5). In each group, the collected spinal cord tissue was thawed, quantitatively weighed, and homogenized immediately according to published methods [41]. The levels of TNF-*α*, IL-1*β*, and IL-6 of the spinal cord supernatant were detected using an ELISA kit (R&D Systems, Minneapolis, MN, USA) in accordance with the kit instructions.

### 2.11. The Effect of H_2_-BMSCs on Spinal Cord Oxidative Stress Detected via Superoxide Dismutase (SOD) and Malondialdehyde (MDA) Kits

To confirm the effect of H_2_/BMSCs on spinal cord oxidative stress, injured spinal cord tissue was collected and subjected to the SOD and MDA kits 3 and 28 days after SCI. The central area of the injured spinal cord tissue (100 mg) was homogenized immediately according to the testing instructions. The SOD activity (U/mg) and MDA concentration (mol/mg protein) of the injured spinal cord were measured by using SOD and MDA kits (Jiancheng Bioengineering Institute, Nanjing, China) according to the testing instructions.

### 2.12. Statistical Analysis

SPSS 21 software (IBM, Chicago, IL, USA) and GraphPad Prism 5 software (GraphPad Software, Inc., La Jolla, CA, USA) were used for data analysis and graphing, respectively. Continuous variables are presented as the mean ± SD. The mean values were compared by using repeated-measure ANOVA and Fisher's LSD post hoc test. Differences were considered statistically significant at *P* < 0.05.

## 3. Result

### 3.1. Characterization of BMSCs

**Figure 1:**
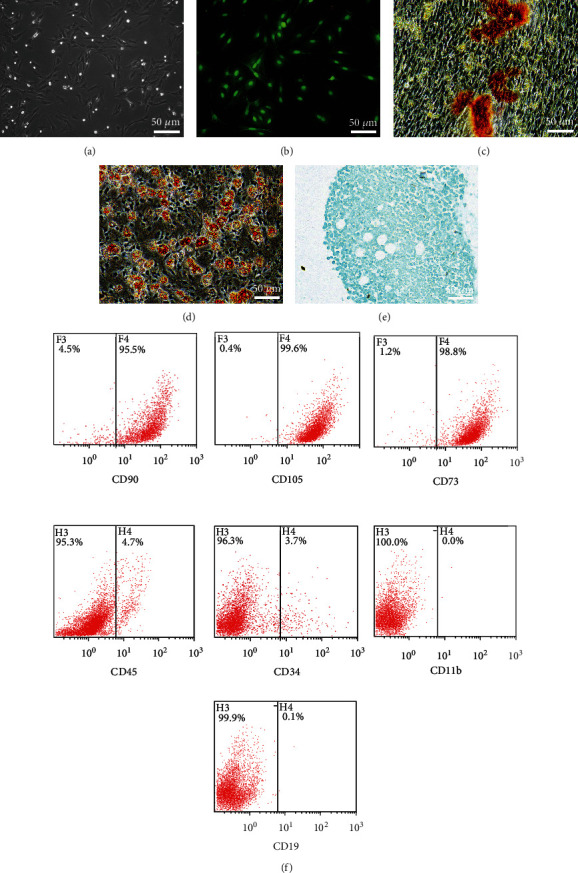
The characterization of BMSCs. (a) BMSCs were observed using a microscope. BMSC-GFP cells showed green fluorescence (b) under a fluorescence microscope. (c–e) The result of BMSCs cultured in osteogenic, lipogenic, and chondrogenic media for 3 weeks. (f) The rates of CD90, CD105, CD73, CD45, CD34, CD11b, and CD19 positivity on P3 BMSCs.

The morphology of BMSCs was observed under a light microscope, which showed that the cells were uniform in shape, with a spindle shape, and the cell purity was high (Figure 1(a)). BMSC-GFP showed strong green fluorescence under a fluorescence microscope (Figure 1(b)). After a 3-week culture, the purified BMSCs exhibited osteogenic, lipogenic, and chondrogenic differentiation abilities (Figures 1(c)–1(e)). Flow cytometry showed that the positive rates of CD90, CD105, and CD73 in P3 BMSCs were higher than 95%, and those of CD45, CD34, CD11b, and CD19 were lower than 4%, indicating that the obtained P3 BMSCs were of high purity (Figure 1(f)). The P3 BMSCs obtained in the experiment were of high purity and showed strong green fluorescence, implying that they can be used for subsequent experiments.

### 3.2. H_2_ Promotes the Proliferation Ability of BMSCs

**Figure 2:**
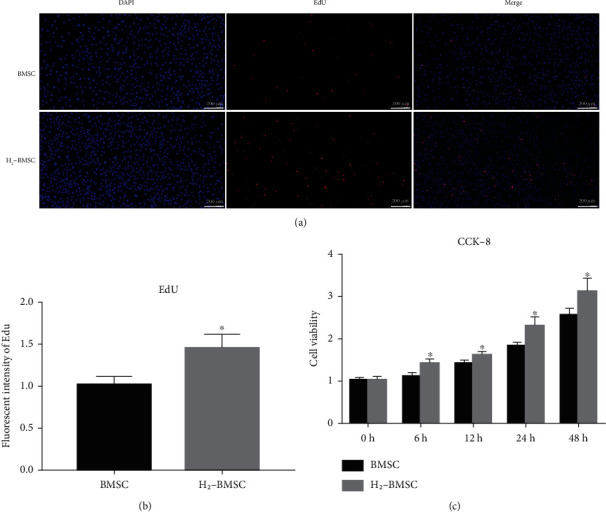
H_2_ promotes the proliferation ability of BMSCs. (a) The proliferation of BMSCs detected by using EdU staining. (b) Immunofluorescence cell count in EdU detection. (c) Cell proliferation after 0, 6, 12, 24, and 48 h detected by using CCK-8. Data as mean ± SD, ^∗^*P* < 0.05 vs. BMSC group (*n* = 6).

The results of CCK-8 and EdU staining showed that H_2_ could significantly promote the proliferation ability of BMSCs. BMSCs were cultured for 6, 12, 24, and 48 h under HRM conditions. The CCK-8 kit was used to detect the proliferation of BMSCs, which showed that the cell proliferation in the H_2_-BMSC group was significantly higher than that in the BMSC group at 6, 12, 24, and 48 h (Figure 2, *P* < 0.05). The results of EdU staining showed that when BMSCs were cultured in HRM for 48 h, the cell proliferation in the H_2_-BMSC group (1.45 ± 0.16) was significantly increased compared with that in the BMSC group (1.01 ± 0.10, *P* < 0.05). These results suggested that H_2_ can promote the BMSC proliferation ability in vitro.

### 3.3. H_2_ Promotes the Migration Ability of BMSCs

**Figure 3:**
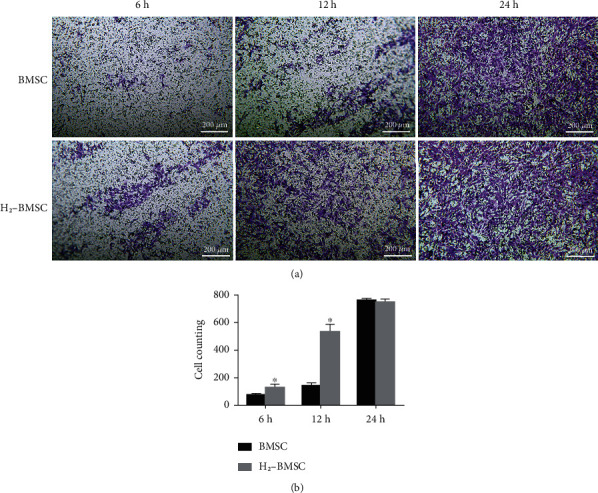
H_2_ promotes the migration ability of BMSCs. (a) shows the cell migration in the BMSC and H_2_-BMSC groups at 6 h (73.20 ± 10.43 vs. 158.60 ± 22.11), 12 h (140.60 ± 22.69 vs. 533.20 ± 53.68), and 24 h (761.80 ± 13.27 vs. 747.80 ± 24.03). (b) shows the number of migrated BMSC in the two groups at different times. Data as the mean ± SD. ^∗^*P* < 0.05 vs. BMSC group (*n* = 6).

H_2_ significantly promoted BMSC migration. The results of Transwell cell migration experiments showed that with the extension of the culture time, more cells migrated. After 6 and 12 h of culture, the number of cells passing through the membrane in the H_2_-BMSC group increased significantly compared with that in the BMSC group (Figure 3, *P* < 0.05). After 24 h of culture, the number of cells that passed through the membrane in both groups was greater, and the difference was not statistically significant (*P* > 0.1), indicating that most of the cells in the two groups had passed through the membrane after 24 h of culture. H_2_ significantly increased the migration ability of BMSCs.

### 3.4. H_2_ Increases BMSC Tolerance to SDM as Detected via Flow Cytometry

**Figure 4:**
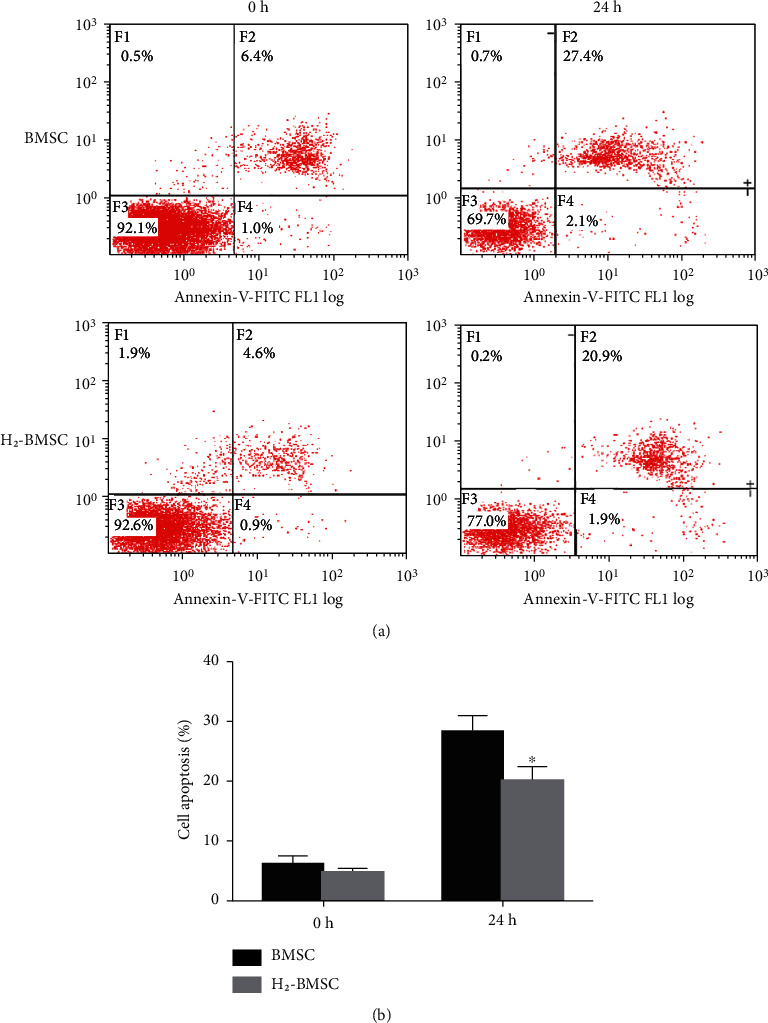
H_2_ increases the BMSC tolerance to SDM as detected via flow cytometry. (a) presents the apoptosis rate of BMSCs detected via flow cytometry after tolerance to SDM for 0 and 12 h in the BMSC and H_2_-BMSC groups. (b) shows the apoptosis rate in the BMSC and H_2_-BMSC groups. Data as the mean ± SD. ^∗^*P* < 0.05 vs. BMSC group (*n* = 6).

BMSC tolerance to SDM was reflected by cell apoptosis detected via flow cytometry. The apoptosis rate was calculated as the early apoptosis plus late apoptosis rate. The results of flow cytometry showed that both groups had lower apoptosis before serum-free culture, and there was no statistical difference (Figure 4, 6.04 ± 1.53 vs. 4.74 ± 0.69, *P* > 0.05). After 24 h of culture in serum-free conditions, the apoptosis rate of the BMSC group was significantly higher than that of the H_2_-BMSC group (28.20 ± 2.66 vs. 20.08 ± 2.47, *P* < 0.05). The FCM test results showed that H_2_ was sufficient to decrease the BMSC apoptosis rate in response to serum deprivation for 24 h. BMSCs cultured in HRM showed much higher tolerance to SDM.

### 3.5. H_2_ Enhances the Effect of BMSC Transplantation in the Treatment of Motor Function in the Rat Hindlimb after SCI

**Figure 5:**
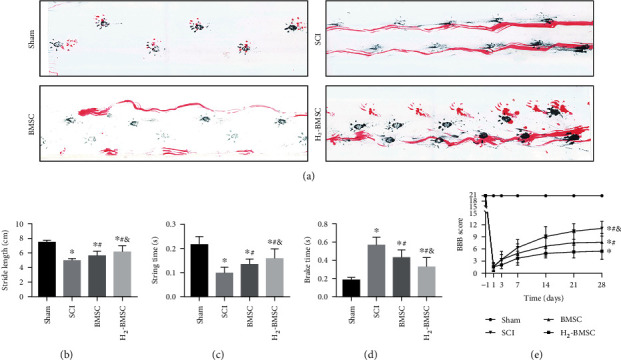
H_2_ enhances the effect of BMSC transplantation in the treatment of motor function after SCI. (a) shows footprint and walking images of each group 28 days after SCI (blue ink: forelimb prints; red: hindlimb prints). (b–d) show the quantitative analysis of stride length, brake time, and stride time of each group 28 days after SCI. (e) shows the BBB score performed 1 d before surgery and 1 d, 3 d, 7 d, 14 d, 21 d, and 28 d after surgery. Data as mean ± SD. ^∗^*P* < 0.05 vs. the sham group. ^#^*P* < 0.05 vs. the SCI group. ^&^*P* < 0.05 vs. the BMSC group (*n* = 10).

The BBB scale, footprint analysis, and CatWalk gait analysis (Supplementary video 1) were used to investigate the effect of H_2_/BMSC in the treatment of motor function in rat hindlimbs after SCI. Footprint analysis and CatWalk gait analysis were performed 28 days after SCI. The BBB scores were assessed before and after 1, 3, 7, 10, 14, 21, and 28 days of SCI. The BBB score was notably lower in the SCI, BMSC, and H_2_-BMSC groups than in the sham group after SCI (*P* < 0.05). Rats that received H_2_-BMSC had significantly higher BBB scores than those in the BMSC group 14, 21, and 28 days after SCI (Figure 5(e), *P* < 0.05). The rats in the BMSC groups showed relatively continuous stumbling of the hindlimbs (red ink), while rats in the H_2_-BMSC group were able to partly move the joints of the hindlimbs and walked with discontinuous trajectories (Figure 5(a)). Although it was shorter than that of the sham group, the stride length of the H_2_-BMSC group was significantly longer than that of the SCI and BMSC groups (Figure 5(b)). The striding time of the BMSC and H_2_-BMSC groups was significantly longer than that of the SCI group but shorter than that of the sham group. Compared with the BMSC group, the striding time of the H_2_-BMSC group was much longer (Figure 5(c), *P* < 0.05). The brake time showed the reverse trend among these groups, as shown in Figure 5(d). The BBB scale, footprint analysis, and CatWalk gait analysis indicated that H_2_ enhances the effect of BMSC transplantation in the treatment of motor function in the rat hindlimb after SCI.

### 3.6. Histopathological Analysis

**Figure 6:**
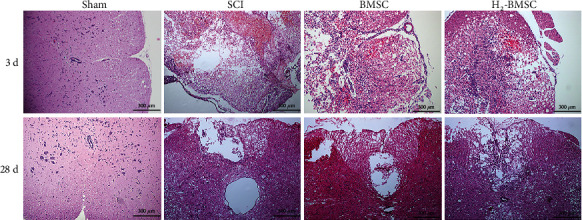
HE staining of H_2_-BMSCs 3 and 28 days after SCI.

The results of HE staining showed that the boundary between the gray matter and white matter of the spinal cord in the SCI, BMSC, and H_2_-BMSC groups was unclear, with hemorrhage, liquefaction, inflammatory cell infiltration, nerve fiber disorder, and neuronal necrosis and atrophy 3 days after SCI. In the H_2_-BMSC group, the amounts of both neuronal cells and inflammatory cells were less than those in the BMSC group (Figure 6). 28 days after SCI, syringomyelia was observed in the SCI group, with unclear boundaries between the gray and the white matter and infiltration of inflammatory cells. The syringomyelia was smaller, and inflammatory cell infiltration was less in the BMSC and H_2_-BMSC groups (Figure 6). BMSC transplantation can significantly reduce the pathological injury of the spinal cord at the SCI site. Compared with the BMSC group, H_2_-BMSCs had less cell death, less bleeding, and less inflammatory cell infiltration. The histopathological analysis showed that H_2_ can significantly enhance the ability of BMSC transplantation to repair spinal cord tissue in SCI rats.

### 3.7. H_2_ Enhances the Migration and Survival of BMSCs after Transplantation

**Figure 7:**
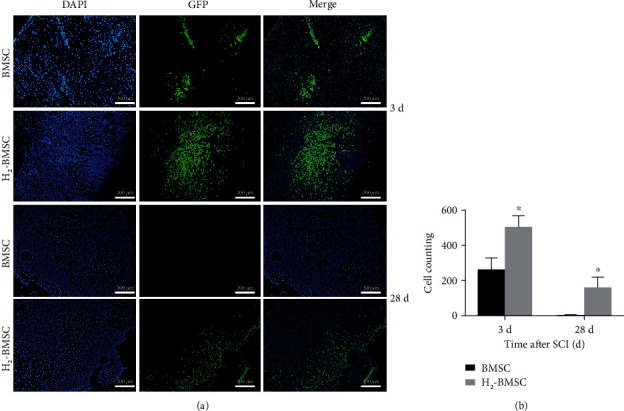
H_2_ enhanced the migration and survival of BMSCs after transplantation. (a) shows green fluorescence of transplanted BMSCs in spinal cord tissue under a fluorescence microscope. (b) shows the count of GFP-positive cells. Data as mean ± SD, ^∗^*P* < 0.05 vs. the BMSC group (*n* = 5).

Three and 28 days after SCI, the number and distribution of BMSC-GFP in the spinal cord were observed using immunofluorescence staining (Figure 7). GFP positivity was used to identify transplanted BMSC-GFP. Immunofluorescence results showed that there were no green fluorescent cells in either the sham group or the SCI group. Three days after cell transplantation, green fluorescent cells were observed in both the BMSC group and the H_2_-BMSC group, and the number of green fluorescent cells in the H_2_-BMSC group (501.60 ± 67.32) was much higher compared with that in the BMSC group (259.80 ± 68.42). After 28 days of cell transplantation, no green fluorescent cells were found in the BMSC group (0), but green fluorescent cells were still visible in the H_2_-BMSC group (156.60 ± 62.61). H_2_ significantly increased the number of BMSCs in the injured spinal cord (Figure 7, *P* < 0.05). On the one hand, the possible reason is that H_2_ can enhance the anti-injury ability of transplanted BMSCs, allowing them to survive better in the injured spinal cord, and on the other hand, it enhances the migration ability of transplanted BMSCs, allowing more transplanted cells to enter the injured spinal cord.

### 3.8. H_2_ Can Enhance the Ability of BMSCs to Suppress Inflammation in SCI

**Figure 8:**
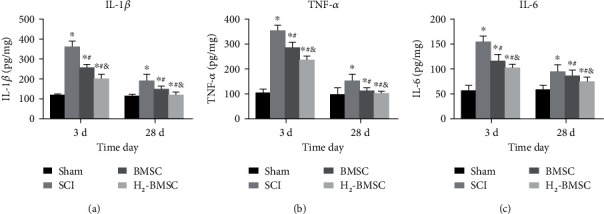
Determining the content of IL-1*β* (a), TNF-*α* (b), and IL-6 (c) via ELISA. Data as mean ± SD. ^∗^*P* < 0.05 vs. the sham group. ^#^*P* < 0.05 vs. the SCI group. ^&^*P* < 0.05 vs. the BMSC group (*n* = 5).

The levels of IL-1*β*, TNF-*α*, and IL-6 were detected at 3 and 28 days after SCI via ELISA (Figure 8). The levels of TNF-*α*, IL-1*β*, and IL-6 in the SCI group increased significantly compared with those in the sham group (*P* < 0.05). The TNF-*α*, IL-1*β*, and IL-6 content in the BMSC and H_2_-BMSC groups were much lower than that in the SCI group (*P* < 0.05). In addition, the levels of TNF-*α*, IL-1*β*, and IL-6 decreased significantly in the H_2_-BMSC group compared with the BMSC group (*P* < 0.05). All these results indicated that H_2_ can enhance the ability of BMSCs to suppress inflammation in SCI.

### 3.9. H_2_ Can Enhance the Ability of BMSCs to Suppress Oxidative Stress in SCI

**Figure 9:**
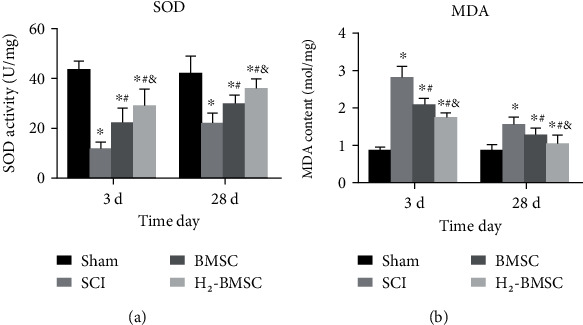
Determining the spinal cord oxidative stress of SOD (a) and MDA (b). Data as mean ± SD. ^∗^*P* < 0.05 vs. the sham group. ^#^*P* < 0.05 vs. the SCI group. ^&^*P* < 0.05 vs. the BMSC group (*n* = 5).

The content of MDA and SOD reflects the level of spinal cord oxidative stress. The levels of MDA and SOD were measured 3 and 28 days after SCI (Figure 9). After 3 and 28 days, the SOD activity at the injury site of SCI rats was significantly decreased, and the MDA content was increased significantly, especially 3 days after SCI. Compared with the SCI group, the SOD activity was significantly increased, and the MDA level was significantly decreased in BMSC and H_2_-BMSC after 3 and 28 days (*P* < 0.05). In addition, compared with the BMSC group, MDA decreased significantly, and SOD activity increased significantly in the H_2_-BMSC group (*P* < 0.05). H_2_ markedly enhanced the ability of BMSCs to suppress oxidative stress in SCI after SCI.

## 4. Discussion

This study was aimed at investigating the hypothesis that H_2_ can promote BMSC transplantation in the treatment of SCI in rats. Our study confirmed that BMSC transplantation had a good effect on the treatment of SCI, which was consistent with published studies [42, 43]. In addition, we found that H_2_ can significantly enhance the therapeutic effect of BMSCs on SCI. This study confirmed that H_2_ promotes the therapeutic effect of BMSC transplantation in the treatment of SCI in rats. It provides a simple and effective measure for stem cell transplantation in the treatment of SCI.

The treatment of SCI is still a worldwide problem [44, 45]. Studies have shown that excessive production of free radicals, such as reactive oxygen species (ROS) in the early stage of SCI, can lead to oxidative damage to DNA, lipids, and proteins, resulting in 8-hydroxydeoxyguanosine (8-OHdG), 4-hydroxynonenal (4-HNE), and nitrotyrosine (NTY) [46]. In the early inflammatory response stage of SCI, a large number of microglia proliferate and activate, leading to the occurrence of an inflammatory cascade, resulting in the occurrence of secondary SCI [47]. Changes in the spinal cord microenvironment may not only cause further damage to the spinal cord but also lead to low viability of transplanted cells and limited therapeutic effects. The key to the recovery of the nervous system function is the survival of nerve cells and the regeneration of axons. Reynolds et al. extracted neural stem cells with differentiation potential from rat nerve tissue and applied them to the treatment of SCI, bringing hope to the treatment of SCI [48]. At present, most animal experiments have shown that the acute phase of SCI is the best period for stem cell transplantation [18, 19]. In clinical trials, some scholars found that the effect of BMSCs on acute and subacute SCI was significantly better than that on chronic SCI [49]. The special microenvironment in the acute phase of SCI can not only aggravate the degree of SCI but also lead to problems such as a low survival rate of transplanted stem cells, insufficient secretion, and poor directional differentiation ability [16]. At present, there are many ways to treat SCI with BMSCs, including intralesional transplantation, ICT, and intravenous transplantation [50]. Clinical trials have confirmed the safety of the clinical transplantation cells via ICT [51, 52]. Though the more effective therapy for cell transplantation is ICT, the effectiveness of delivery cells to the injured lesion is 4.1% at 4 days and drops to 3.4% at 21 days [53]. The reasons for the low survival rates postadministration of the transplanted cells remain unclear. Multiple factors affect cell survival, including inflammation, oxidative stress, immune reactions of the host, and the interplay with grafted cells [54]. Studies have shown that early engraftment cell survival can be significantly improved by reducing inflammation and oxidative stress in the SCI area [55]. The effect of immune reactions of the host, the interplay of grafted cells with transplanted cells, and the effect of H_2_ on local immune responses need to be further investigated [50, 56].

Different scholars have made many attempts to solve the problems of the low survival rate of transplanted cells and the poor therapeutic effect. Our previous study found that hypoxic preconditioning can increase the effects of BMSC on SCI in rats [17]. Luo et al. studied the cotransplantation of neural stem cells and olfactory ensheathing cells and found that cotransplantation could promote the survival of neural stem cells and improve hyperalgesia [57]. Gong et al. found that the transfection of BMSCs through lentivirus-mediated neurotrophic factor 3 can significantly improve the effect of cell transplantation in the treatment of SCI [58]. Yazdani et al. achieved a certain effect through the combined transplantation of autologous Schwann cells and BMSCs in the treatment of chronic SCI [59]. Although these methods have enhanced the effect of cell transplantation in the treatment of SCI to a certain extent, there are problems such as complicated operations and unsatisfactory effects. The present study combined H_2_ with BMSC strategies in experiments to enhance the survival rate, migration, and neurological recovery of transplanted cells.

H_2_ is a common small-molecule gas in nature. There are hydrogen-producing bacteria in the human intestine. In the past, it was believed that hydrogen could not react biologically with organisms in the body [60]. In 2007, Ohsawa et al. found that hydrogen can selectively scavenge hydroxyl radicals (·OH) and peroxidative nitrate anions (ONOO-), showing a strong antioxidant effect, which started the upsurge of research on hydrogen in medicine [22]. Unlike most well-known antioxidants, the key of H_2_'s role is to neutralize free radicals, which has the following advantages: (1) H_2_ has a selective antioxidant effect and does not affect the molecular action through signaling mechanisms, (2) H_2_ itself and its reaction products are nontoxic, and (3) H_2_ can easily pass through the blood-brain barrier and penetrate deep into the cell. H_2_ not only plays an antioxidant role by scavenging free radicals but also has anti-inflammatory and antiapoptotic effects [61–63]. At present, the administration methods of H_2_ to treat diseases mainly include inhalation of H_2_ [64], HRS injection [62, 65–67], and drinking HRS. Among them, H_2_ inhalation was the first to be used, but because H_2_ is flammable and explosive, there are certain risks, and the requirements for equipment are relatively high, which limits the application of this method [64]. In animal studies, intraperitoneal injection of HRS is more commonly used because of its convenient application and controllable dose. H_2_ therapy has been successful in various animal models of ischemia–reperfusion injury, Alzheimer's disease, carbon monoxide toxicity, delayed encephalopathy, and atherosclerosis [23–25]. Therefore, this experiment also adopted the way of intraperitoneal injection of HRS. H_2_ has been widely used in the treatment of SCI with good results [26–30, 68, 69]. To solve the problem of the low local concentrations of hydrogen, we designed a pH-responsive delivery of H_2_ to treat SCI and achieved good results [30]. The mechanism of H_2_ in the treatment of SCI is mainly to improve the local microenvironment of the injured spinal cord and to reduce the local inflammatory response, oxidative stress, and glial scar formation. However, H_2_ has no obvious regeneration effect on damaged neurons and axons. Therefore, in this experiment, the method of using H_2_ combined with stem cell transplantation to treat SCI achieved a good therapeutic effect and greatly prolonged the survival time and quantity of transplanted cells in the spinal cord.

There are several deficiencies in this experiment. First, many studies have shown the therapeutic effect of hydrogen on SCI, but the mechanism of action is unclear. The specific molecular mechanisms involved in this process require further basic research. Second, rats were used in this study because of their maturity, and the operation is simple, safe, and reproducible with the rat SCI-modeling technology. However, there are differences between humans and rats. Further studies are needed to confirm the therapeutic effect of H_2_-BMSC in humans. Third, whether H_2_ promotes the effectiveness of BMSC transplantation in rats with SCI in a dose-dependent manner needs to be further explored. The reason we did not perform the H_2_ dose-dependent assay in the cellular experiments was that HRM with different H_2_ concentrations was difficult to prepare and could not be precisely controlled, resulting in experimental error. We speculated that H_2_ promoted the proliferation and migration ability of BMSCs in a dose-dependent manner. However, the conclusions need to be proven by further experiments.

In conclusion, we reported a novel strategy to treat SCI by combining hydrogen and BMSC transplantation. Hydrogen can enhance the migration and proliferation of BMSCs to repair SCI. Hydrogen and MSC codelivery is an effective and simple method to improve BMSC transplantation in the treatment of SCI.

## Data Availability

The datasets used and/or analyzed during the current study are available from the corresponding author on reasonable request.

## Supplementary Materials

Supplementary Materials Supplementary video 1: the walk video of the rats after SIC recorded via CatWalk gait analysis.

## References

[1] Chen C., Qiao X., Liu W., Fekete C., Reinhardt J. D. (2022). Epidemiology of spinal cord injury in China: a systematic review of the Chinese and English literature. *Spinal Cord*.

[2] Barbiellini Amidei C., Salmaso L., Bellio S., Saia M. (2022). Epidemiology of traumatic spinal cord injury: a large population-based study. *Spinal Cord*.

[3] Kwon B. K., Tetzlaff W., Grauer J. N., Beiner J., Vaccaro A. R. (2004). Pathophysiology and pharmacologic treatment of acute spinal cord injury. *The Spine Journal*.

[4] Tabarés Seisdedos R. (2019). Global, regional, and national burden of traumatic brain injury and spinal cord injury, 1990-2016: a systematic analysis for the Global Burden of Disease Study 2016. *Lancet Neurology*.

[5] Ashammakhi N., Kim H. J., Ehsanipour A. (2019). Regenerative therapies for spinal cord injury. *Tissue Engineering. Part B, Reviews*.

[6] Guo S., Redenski I., Levenberg S. (2021). Spinal cord repair: from cells and tissue engineering to extracellular vesicles. *Cell*.

[7] Liu X., Hao M., Chen Z. (2021). 3D bioprinted neural tissue constructs for spinal cord injury repair. *Biomaterials*.

[8] Cofano F., Boido M., Monticelli M. (2019). Mesenchymal stem cells for spinal cord injury: current options, limitations, and future of cell therapy. *International Journal of Molecular Sciences*.

[9] Gazdic M., Volarevic V., Harrell C. R. (2018). Stem cells therapy for spinal cord injury. *International Journal of Molecular Sciences*.

[10] Gao L., Peng Y., Xu W. (2020). Progress in stem cell therapy for spinal cord injury. *Stem Cells International*.

[11] Curtis E., Martin J. R., Gabel B. (2018). A first-in-human, phase I study of neural stem cell transplantation for chronic spinal cord injury. *Cell Stem Cell*.

[12] Gong Z., Xia K., Xu A. (2020). Stem cell transplantation: a promising therapy for spinal cord injury. *Current Stem Cell Research & Therapy*.

[13] Fan B., Wei Z., Yao X. (2018). Microenvironment imbalance of spinal cord injury. *Cell Transplantation*.

[14] Fleming J. C., Norenberg M. D., Ramsay D. A. (2006). The cellular inflammatory response in human spinal cords after injury. *Brain: A Journal of Neurology*.

[15] Eghwrudjakpor P. O., Allison A. B. (2010). Oxidative stress following traumatic brain injury: enhancement of endogenous antioxidant defense systems and the promise of improved outcome. *Nigerian Journal of Medicine*.

[16] Su M., Guan H., Zhang F., Gao Y., Teng X., Yang W. (2016). HDAC6 regulates the chaperone-mediated autophagy to prevent oxidative damage in injured neurons after experimental spinal cord injury. *Oxidative Medicine and Cellular Longevity*.

[17] Wang W., Huang X., Lin W. (2018). Hypoxic preconditioned bone mesenchymal stem cells ameliorate spinal cord injury in rats via improved survival and migration. *International Journal of Molecular Medicine*.

[18] Yan K., Zhang R., Sun C. (2013). Bone marrow-derived mesenchymal stem cells maintain the resting phenotype of microglia and inhibit microglial activation. *PLoS One*.

[19] Yang X., Hao D., Zhang H., Liu B., Yang M., He B. (2017). Treatment with hydrogen sulfide attenuates sublesional skeletal deterioration following motor complete spinal cord injury in rats. *Osteoporosis International*.

[20] Huang L., Fu C., Xiong F., He C., Wei Q. (2021). Stem cell therapy for spinal cord injury. *Cell Transplantation*.

[21] Hu X.-C., Lu Y.-B., Yang Y.-N. (2021). Progress in clinical trials of cell transplantation for the treatment of spinal cord injury: how many questions remain unanswered?. *Neural Regeneration Research*.

[22] Ohsawa I., Ishikawa M., Takahashi K. (2007). Hydrogen acts as a therapeutic antioxidant by selectively reducing cytotoxic oxygen radicals. *Nature Medicine*.

[23] Bai Y., Han Q., Dong B. (2022). PPAR*α* contributes to the therapeutic effect of hydrogen gas against sepsis- associated encephalopathy with the regulation to the CREB-BDNF signaling pathway and hippocampal neuron plasticity-related gene expression. *Brain Research Bulletin*.

[24] Wu Y., Yuan M., Song J., Chen X., Yang H. (2019). Hydrogen gas from inflammation treatment to cancer therapy. *ACS Nano*.

[25] Kou Z., Zhao P., Wang Z. (2019). Acid-responsive H_2_-releasing Fe nanoparticles for safe and effective cancer therapy. *Journal of Materials Chemistry B*.

[26] Chen C., Chen Q., Mao Y. (2010). Hydrogen-rich saline protects against spinal cord injury in rats. *Neurochemical Research*.

[27] Chen X., Cui J., Zhai X. (2018). Inhalation of hydrogen of different concentrations ameliorates spinal cord injury in mice by protecting spinal cord neurons from apoptosis, oxidative injury and mitochondrial structure damages. *Cellular Physiology and Biochemistry*.

[28] Huang Y., Xie K., Li J. (2011). Beneficial effects of hydrogen gas against spinal cord ischemia-reperfusion injury in rabbits. *Brain Research*.

[29] Kimura A., Suehiro K., Mukai A. (2022). Protective effects of hydrogen gas against spinal cord ischemia-reperfusion injury. *The Journal of Thoracic and Cardiovascular Surgery*.

[30] Liu Y., Wang Y., Xiao B. (2021). pH-responsive delivery of H_2_ through ammonia borane-loaded mesoporous silica nanoparticles improves recovery after spinal cord injury by moderating oxidative stress and regulating microglial polarization. *Regenerative Biomaterials*.

[31] Yao L., Chen H., Wu Q., Xie K. (2019). Hydrogen-rich saline alleviates inflammation and apoptosis in myocardial I/R injury via PINK-mediated autophagy. *International Journal of Molecular Medicine*.

[32] Li J., Wang C., Zhang J. H., Cai J.-M., Cao Y.-P., Sun X.-J. (2010). Hydrogen-rich saline improves memory function in a rat model of amyloid-beta- induced Alzheimer's disease by reduction of oxidative stress. *Brain Research*.

[33] Deng G., Wang W., Yang C., Gao R., Yang X., Ye X. (2016). Shaking improves the whole bone marrow adherent method of purification. *Molecular Medicine Reports*.

[34] Lee R. H., Kim B., Choi I. (2004). Characterization and expression analysis of mesenchymal stem cells from human bone marrow and adipose tissue. *Cellular Physiology and Biochemistry*.

[35] Cy M., Xue F., Zq Z., Hao T., Sb G., Feng W. (2020). Influence of microRNA-141 on inhibition of the proliferation of bone marrow mesenchymal stem cells in steroid-induced OsteonecrosisviaSOX11. *Orthopaedic Surgery*.

[36] Khan T., Havey R. M., Sayers S. T., Patwardhan A., King W. W. (1999). Animal models of spinal cord contusion injuries. *Laboratory Animal Science*.

[37] Falconer J. C., Narayana P. A., Bhattacharjee M., Liu S. J. (1996). Characterization of an experimental spinal cord injury model using waveform and morphometric analysis. *Spine*.

[38] Paul C., Samdani A. F., Betz R. R., Fischer I., Neuhuber B. (2009). Grafting of human bone marrow stromal cells into spinal cord injury: a comparison of delivery methods. *Spine*.

[39] Basso D. M., Beattie M. S., Bresnahan J. C. (1996). Graded histological and locomotor outcomes after spinal cord contusion using the NYU weight-drop device versus transection. *Experimental Neurology*.

[40] Hamers F. P., Koopmans G. C., Joosten E. A. (2006). CatWalk-assisted gait analysis in the assessment of spinal cord injury. *Journal of Neurotrauma*.

[41] Wang C. X., Olschowka J. A., Wrathall J. R. (1997). Increase of interleukin-1*β* mRNA and protein in the spinal cord following experimental traumatic injury in the rat. *Brain Research*.

[42] Damianakis E. I., Benetos I. S., Evangelopoulos D. S., Kotroni A., Vlamis J., Pneumaticos S. G. (2022). Stem cell therapy for spinal cord injury: a review of recent clinical trials. *Cureus*.

[43] Saini R., Pahwa B., Agrawal D. (2022). Efficacy and outcome of bone marrow derived stem cells transplanted via intramedullary route in acute complete spinal cord injury - A randomized placebo controlled trial. *Journal of Clinical Neuroscience*.

[44] McDonald J. W., Sadowsky C. (2002). Spinal-cord injury. *The Lancet*.

[45] Eckert M. J., Martin M. J. (2017). Trauma: spinal cord injury. *The Surgical Clinics of North America*.

[46] Tabak O., Gelisgen R., Erman H. (2011). Oxidative lipid, protein, and DNA damage as oxidative stress markers in vascular complications of diabetes mellitus. *Clinical and Investigative Medicine*.

[47] Jung K., Min D. S., Sim K. B. (2003). Upregulation of phospholipase D1 in the spinal cords of rats with clip compression injury. *Neuroscience Letters*.

[48] Reynolds B. A., Weiss S. (1992). Generation of neurons and astrocytes from isolated cells of the adult mammalian central nervous system. *Science*.

[49] Lu Y., Zhang W., Tian Z. (2022). The optimal transplantation strategy of umbilical cord mesenchymal stem cells in spinal cord injury: a systematic review and network meta-analysis based on animal studies. *Stem Cell Research & Therapy*.

[50] Dasari V. R., Veeravalli K. K., Dinh D. H. (2014). Mesenchymal stem cells in the treatment of spinal cord injuries: a review. *World Journal of Stem Cells*.

[51] Saito F., Nakatani T., Iwase M. (2012). Administration of cultured autologous bone marrow stromal cells into cerebrospinal fluid in spinal injury patients: a pilot study. *Restorative Neurology and Neuroscience*.

[52] Suzuki Y., Ishikawa N., Omae K. (2014). Bone marrow-derived mononuclear cell transplantation in spinal cord injury patients by lumbar puncture. *Restorative Neurology and Neuroscience*.

[53] Shin D. A., Kim J. M., Kim H. I. (2013). Comparison of functional and histological outcomes after intralesional, intracisternal, and intravenous transplantation of human bone marrow-derived mesenchymal stromal cells in a rat model of spinal cord injury. *Acta Neurochirurgica*.

[54] Lu P. (2017). Stem cell transplantation for spinal cord injury repair. *Progress in Brain Research*.

[55] Gao S., Ding J., Xiao H.-J. (2014). Anti-inflammatory and anti-apoptotic effect of combined treatment with methylprednisolone and amniotic membrane mesenchymal stem cells after spinal cord injury in rats. *Neurochemical Research*.

[56] Neuhuber B., Himes B. T., Shumsky J. S., Gallo G., Fischer I. (2005). Axon growth and recovery of function supported by human bone marrow stromal cells in the injured spinal cord exhibit donor variations. *Brain Research*.

[57] Luo Y., Zou Y., Yang L. (2013). Transplantation of NSCs with OECs alleviates neuropathic pain associated with NGF downregulation in rats following spinal cord injury. *Neuroscience Letters*.

[58] Gong Y., Wang H., Xia H. (2015). Stable transfection into rat bone marrow mesenchymal stem cells by lentivirus-mediated NT-3. *Molecular Medicine Reports*.

[59] Yazdani S. O., Hafizi M., Zali A. R. (2013). Safety and possible outcome assessment of autologous Schwann cell and bone marrow mesenchymal stromal cell co-transplantation for treatment of patients with chronic spinal cord injury. *Cytotherapy*.

[60] Iida A., Nosaka N., Yumoto T. (2016). The clinical application of hydrogen as a medical treatment. *Acta Medica Okayama*.

[61] Xue J. L., Song G. H., Qin S. C. (2018). Research advances on preventive and therapeutic effects of hydrogen on cardiovascular and cerebrovascular diseases and underlying mechanisms. *Sheng Li Xue Bao*.

[62] Liu Q., Li B., Song Y. (2016). Hydrogen-rich saline protects against mitochondrial dysfunction and apoptosis in mice with obstructive jaundice. *Molecular Medicine Reports*.

[63] Terasaki Y., Terasaki M., Shimizu A. (2021). Protective effects of hydrogen against irradiation. *Current Pharmaceutical Design*.

[64] Shin S. S., Hwang M., Diaz-Arrastia R., Kilbaugh T. J. (2021). Inhalational gases for neuroprotection in traumatic brain injury. *Journal of Neurotrauma*.

[65] Chen X. X., Zhou X. Q., Wei R. L. (2018). Research progress of hydrogen-rich saline for eye diseases. *Zhonghua Yan Ke Za Zhi*.

[66] Chu X., Cao L., Yu Z. (2019). Hydrogen-rich saline promotes microglia M2 polarization and complement-mediated synapse loss to restore behavioral deficits following hypoxia-ischemic in neonatal mice via AMPK activation. *Journal of Neuroinflammation*.

[67] Jiang B., Li Y., Dai W., Wu A., Wu H., Mao D. (2021). Hydrogen-rich saline alleviates early brain injury through regulating of ER stress and autophagy after experimental subarachnoid hemorrhage. *Acta Cirúrgica Brasileira*.

[68] Zhou L., Wang X., Xue W. (2013). Beneficial effects of hydrogen-rich saline against spinal cord ischemia- reperfusion injury in rabbits. *Brain Research*.

[69] Ge L., Wei L. H., Du C. Q. (2017). Hydrogen-rich saline attenuates spinal cord hemisection-induced testicular injury in rats. *Oncotarget*.

